# Host Reproductive Phenology Drives Seasonal Patterns of Host Use in Mosquitoes

**DOI:** 10.1371/journal.pone.0017681

**Published:** 2011-03-07

**Authors:** Nathan D. Burkett-Cadena, Christopher J. W. McClure, Russell A. Ligon, Sean P. Graham, Craig Guyer, Geoffrey E. Hill, Stephen S. Ditchkoff, Micky D. Eubanks, Hassan K. Hassan, Thomas R. Unnasch

**Affiliations:** 1 Department of Entomology and Plant Pathology, Auburn University, Auburn, Alabama, United States of America; 2 Department of Biological Sciences, Auburn University, Auburn, Alabama, United States of America; 3 School of Forestry and Wildlife Sciences, Auburn University, Auburn, Alabama, United States of America; 4 Department of Entomology, Texas A&M University, College Station, Texas, United States of America; 5 Department of Global Health, University of South Florida, Tampa, Florida, United States of America; Global Viral Forecasting Initiative, United States of America

## Abstract

Seasonal shifts in host use by mosquitoes from birds to mammals drive the timing and intensity of annual epidemics of mosquito-borne viruses, such as West Nile virus, in North America. The biological mechanism underlying these shifts has been a matter of debate, with hypotheses falling into two camps: (1) the shift is driven by changes in host abundance, or (2) the shift is driven by seasonal changes in the foraging behavior of mosquitoes. Here we explored the idea that seasonal changes in host use by mosquitoes are driven by temporal patterns of host reproduction. We investigated the relationship between seasonal patterns of host use by mosquitoes and host reproductive phenology by examining a seven-year dataset of blood meal identifications from a site in Tuskegee National Forest, Alabama USA and data on reproduction from the most commonly utilized endothermic (white-tailed deer, great blue heron, yellow-crowned night heron) and ectothermic (frogs) hosts. Our analysis revealed that feeding on each host peaked during periods of reproductive activity. Specifically, mosquitoes utilized herons in the spring and early summer, during periods of peak nest occupancy, whereas deer were fed upon most during the late summer and fall, the period corresponding to the peak in births for deer. For frogs, however, feeding on early- and late-season breeders paralleled peaks in male vocalization. We demonstrate for the first time that seasonal patterns of host use by mosquitoes track the reproductive phenology of the hosts. Peaks in relative mosquito feeding on each host during reproductive phases are likely the result of increased tolerance and decreased vigilance to attacking mosquitoes by nestlings and brooding adults (avian hosts), quiescent young (avian and mammalian hosts), and mate-seeking males (frogs).

## Introduction

Seasonal patterns of host selection by arthropods are a critical component in the amplification and spillover of arthropod-borne zoonoses [1]. Mosquito-borne viruses, for example, circulate in populations of vertebrate reservoir hosts in annual periods of pre-epidemic virus amplification [2]. This period of virus amplification usually occurs in spring and early summer, when mosquitoes feed predominantly on birds [3], which are the natural reservoir hosts of pathogenic viruses such as West Nile virus, eastern equine encephalitis virus and St. Louis encephalitis virus [4]. Transmission of mosquito-borne viruses to humans usually occurs in late summer and early fall, when the mosquito population exhibits a shift in host use from birds to mammals [1], [3].

Although several hypotheses regarding seasonal shifts in host use by mosquitoes have been proposed and tested, the mechanism underlying these shifts remains elusive. The hypothesis that host-feeding patterns are due to seasonal changes in the abundance of avian hosts was not supported by field data from Florida [3]. A second hypothesis, suggesting that host preference (olfactory attraction) changes as the season progresses, was also not supported by field data [5]. Likewise, no evidence could be found to support a third hypothesis, which proposed that feeding patterns are the result of seasonal changes in the density and feeding success of mosquitoes [6]. A fourth hypothesis, that host-feeding patterns are due to seasonal changes in habitat use by mosquitoes (mosquitoes forage more often in open habitats when humidity is high and are more likely to encounter hosts in open habitats), was indirectly supported by field evidence [5], but the authors neglected to demonstrate that mammals were more abundant in open habitats during times of mosquito foraging. All of the above studies [3], [5], [6] disregard differences in the biology of each host and generalize about mosquito feeding behavior on birds and mammals. While the mosquitoes in these studies were found to feed on a diverse array of birds and mammals, only a few of the host species (3–5) comprised the great majority of mosquito blood meals. It therefore seems more plausible that seasonal patterns of host use are driven by mosquitoes feeding upon a few commonly utilized host species, than by mosquitoes selecting the members of the Class Aves or Class Mammalia. Moreover, apart from host abundance, none of the above studies examined the role of host biology in driving seasonal patterns of host use by mosquitoes, despite evidence that host biology affects defensive behaviors [7], a major factor in determining mosquito feeding success [8], [9].

Many of the host species commonly preyed upon by mosquitoes undergo dramatic seasonal variation in behavior, often associated with reproduction. Specifically, animal parents are known to exhibit changes in habitat-use, time-budget, food choice, and detectability at various stages of their reproductive cycle. Therefore, we hypothesize that seasonal changes in host use by mosquitoes reflects the life history of their most commonly utilized hosts, rather than any trait shared by members of a taxonomic class. Furthermore, because of the changes in host behavior associated with reproduction, we predict that seasonal patterns of host use are driven by temporal peaks in the reproductive biology of a few key host species.

## Results

**Figure 1:**
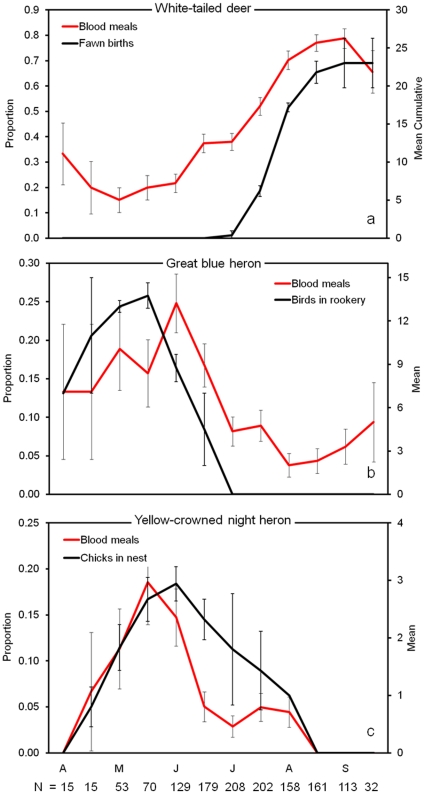
Host reproduction and mosquito parasitism. Seasonal patterns of relative host use (left y axis) and reproductive investment (mean ± SE) in white-tailed deer (a), great blue heron (b), and yellow-crowned night heron (c). Relative host use, the proportion of blood meals (± SE) originating from a given host in a semimonthly period, was determined by PCR-based assays identifying the vertebrate source of blood from field-collected mosquitoes over seven years (2001–2004 and 2006–2008). The number of bloodmeals identified in a period is given on the x axis.

Relative host use was strongly associated with host reproductive biology. The most commonly utilized endothermic hosts were white-tailed deer, great blue heron, and yellow-crowned night heron, which together comprised 75% of blood meals from endothermic hosts. The remaining 25% of (endotherm) bloodmeals were split among 43 other host species (Table S1). While mosquitoes fed on white-tailed deer, great blue herons, and yellow-crowned night herons throughout the year, feeding on each host peaked during birthing/nesting and post-birthing/nesting periods (Figure 1 a–c). For white-tailed deer, the host reproduction (cumulative fawn births) model performed far better than the null model (ΔAIC_c_ = 18.39) with very high likelihood (*w*_i_  =  1) of being the better model (Table 1). Relative feeding on deer peaked in August and September, when the majority (73.0%) of fawns were birthed. For great-blue and yellow-crowned night herons, the host reproduction models (birds in rookery and chicks in nest, respectively) far outperformed the null models, with ΔAIC_c_ values of 6.56 and 15.39, respectively, and very high likelihoods of being the best models (Table 1).

**Table 1:** Model selection statistics for temporal patterns of mosquito host use.

	model	AIC_c_	ΔAIC_c_	*w*_i_
White-tailed deer	host	−9.41	0	1
	null	8.98	18.39	0
Great-blue heron	host	−19.99	0	0.96
	null	−13.42	6.56	0.04
Yellow-crowned night heron	host	−17.3	0	1
	null	−1.91	15.39	0
Frogs	host	11.66	0	0.7
	null	13.36	1.7	0.3

Host models incorporate data on parturition (deer), nest occupancy (herons) and male vocalizations for mate attraction (frogs). See Methods section for details.

**Figure 2:**
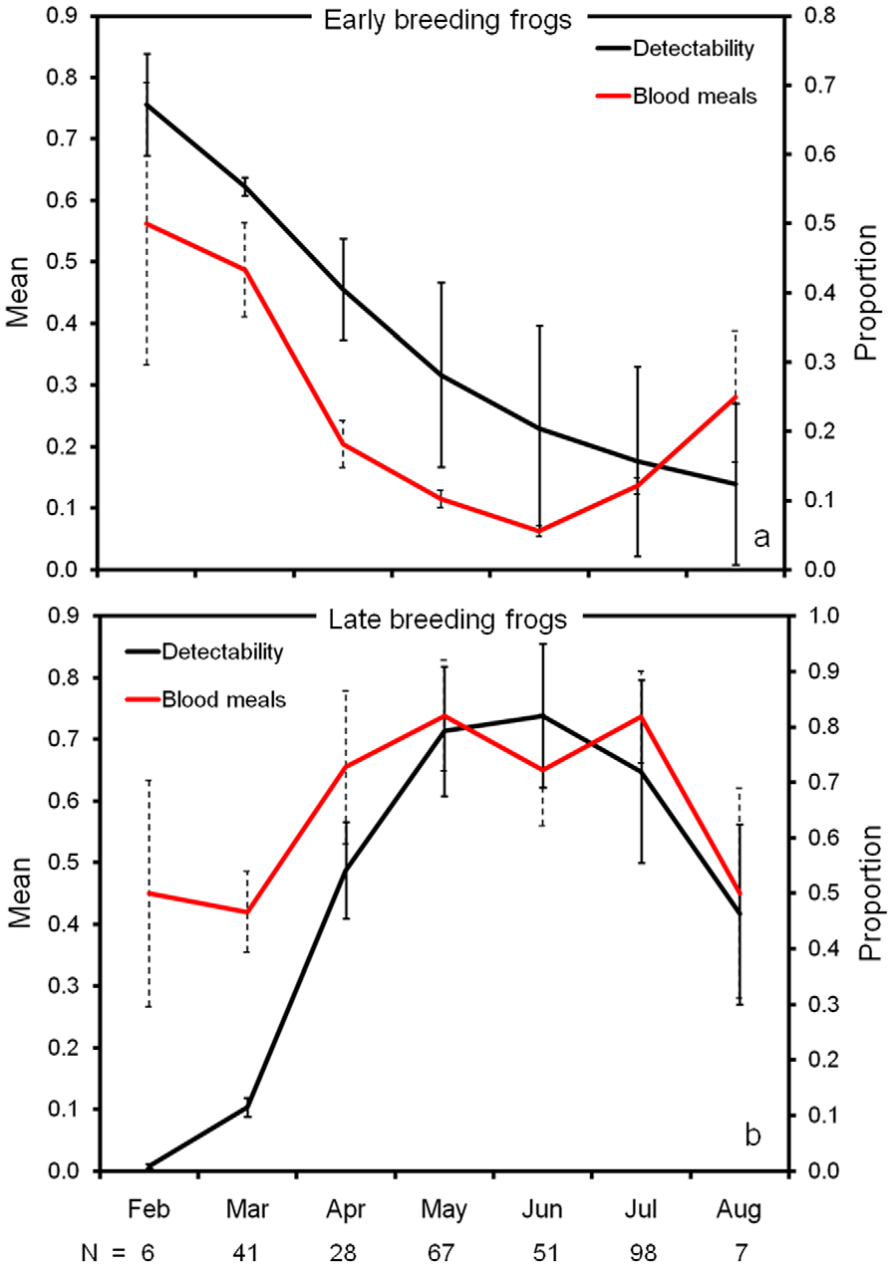
Mate attraction and mosquito parasitism of frogs. Phenology of detectability (vocalizing males) and relative anuran host use by mosquitoes (right y axis). Relative host use, the proportion of blood meals (± SE) originating from (a) early-breeding (Spring Peeper, Leopard frog) and (b) late-breeding (Bullfrog, Green frog, Green treefrog, Grey treefrog, Pine woods treefrog) frogs, was determined by PCR-based assays identifying the vertebrate source of blood from field-collected mosquitoes over seven years (2001–2004 and 2006–2008) from a study site in Alabama, USA. Mean detectability (± SE) was calculated from monthly detection probabilities of each breeding cohort, using general linear mixed models with a binomial distribution and a logit-link. The number of bloodmeals identified in each month is given on the x axis.

For anuran hosts, mosquito feeding on each group was greatest during periods of peak male vocalization for mate attraction. The frog host reproduction model (detectability) was better than the null, although by less than two ΔAIC_c_ (Table 1). Support for the reproductive model was still quite high (*w*_i_  =  0.7). Seasonal host use of early-season (*Pseudacris crucifer* and *Lithobates sphenocephalus*) and late-season breeding frogs (*Hyla chrysoscelis, Hyla cinerea, Hyla femoralis, Lithobates catesbeianus* and *Lithobates clamitans*) paralleled changes in the detectability (Figure 2) of those groups, respectively.

## Discussion

Our data suggest that seasonal changes in patterns of host use by mosquitoes reflect the breeding cycles of their host animals. For endothermic hosts—deer and herons—we found that peaks in host use by mosquitoes were remarkably synchronized with peaks in reproductive investment. Seasonal patterns of mosquito feeding on the ectothermic hosts—frogs—on the other hand, tracked temporal peaks of calling by advertising males. Although mate attraction and post-birth/hatching behavior are quite different components of reproductive investment, both activities likely increase the susceptibility of the respective hosts to being fed upon by mosquitoes.

For animals that exhibit some form of parental care, periods of reproductive investment are likely to increase susceptibility to mosquito attack for both parents and offspring. Brooding mother birds, for example, must stay at the nest to transfer heat to young and protect them from predators [10]. Having to stay in a brooding position on the nest dramatically reduces the frequency and variety of defensive behaviors in which the mother bird can engage, and these defensive behaviors have been shown to greatly influence the feeding success of mosquitoes [8]. Relative feeding on brooding mother birds (versus nestlings) decreases as nestling age increases, suggesting that while brooding, mother birds are more susceptible to attacking mosquitoes [9]. Nestling birds lack the behavioral (e.g., foot stomping and head shaking) and morphological defenses (plumage) exhibited by adult birds, causing nestling birds to be more susceptible to mosquito attack [11]. In fact, the number of defensive movements performed by nestling herons increases with age, resulting in a concomitant decrease in mosquito feeding success [7]. These studies illustrate the various aspects of parental care and offspring development in birds which contribute to variation in mosquito feeding success throughout the nesting period.

As in birds, the reproductive behaviors and developmental stages of mammalian prey items are likely to influence the susceptibility of individuals to mosquito attacks. In deer, age-dependent predator avoidance tactics may expose fawns to increased levels of parasitism from mosquitoes, the incidence of which can be quite high [12]. White-tailed deer fawns, for the first two weeks of life, spend 92% of each day inactive, hiding in vegetation [13]. Our results suggest that this immobility, primarily an anti-predation defense [14], increases the susceptibility of fawns to mosquito parasitism.

For frogs, activities that increase predation risk also contribute to increased parasitism by blood-feeding dipterans. The rate of frog capture by bats is significantly higher when frogs are calling, suggesting that sexual advertisement increases predation risk [15]. The same vocal advertisement is used by some frog-feeding mosquitoes and other blood-sucking flies, which eavesdrop on calling males to locate their hosts [16], [17], [18]. Because calls can be the primary mechanism of mate choice by female frogs and calling is energetically expensive [19], male frogs may devote so much time and energy to calling during the reproductive season that other behaviors, including defensive behaviors, are possibly reduced or abandoned. We infer that a similar mechanism generates the correlation that we observed between male calling activity and use of anurans by mosquitoes.

Various behaviors associated with reproduction have undesirable side effects which expose animals to increased vulnerability to attacks by predators [20]. These behaviors, such as aggregation, display for mate attraction, and caring for young, which increase an animal's risk to predation are also likely to increase an animal's exposure and/or susceptibility to parasite attack [21], resulting in disproportionate feeding on a host during its reproductive phase. Indirect evidence of intense mosquito feeding during the host reproductive season can be found in the temporal ecology of mosquito-transmitted pathogens and parasites. For example, infections of mosquito-transmitted filarial worms in deer peak soon after birth [22], suggesting that vector mosquitoes attack fawns in great numbers during the post-parturition period. In birds, mosquito-borne blood parasites reach their peak parasitemia (parasites/red blood cell) during the breeding season [23], which may reflect intensive mosquito feeding on brooding and nestling birds. These findings further support our own data that indicate that intense feeding on hosts during reproductive phases drive seasonal patterns of relative host use.

While the period of peak mosquito feeding on each host also corresponds to an increase in population size of each species (births/hatching of young), our data do not support the hypothesis that seasonal patterns of host use are driven by increases in host abundance alone. In white-tailed deer, the population increases by approximately two-fold during the period of birthing [24], yet the proportion of bloodmeals derived from deer increases by more than five-fold between May (when deer are fed upon least) and September (maximal feeding on deer). In addition, the increase in deer abundance is short-lived, as only about one to two thirds of fawns survive the first month of life, on average [24], [25]. However, deer continue to be the most important host for mosquitoes through the end of September, at which time mosquito activity abruptly declines. A slight decrease in the proportion of blood meals from deer occurs in late September (Fig 1a) which may correspond to maturation and mobility of fawns, decreasing their susceptibility to attacking mosquitoes. Great blue herons and yellow-crowned night herons (both adults and recently fledged birds) continue to occupy and forage at the site after the nesting period, yet the proportion of blood meals from herons declines sharply after the nesting period. A noticeable increase in bloodmeals derived from great blue herons was observed in September, but does not correspond to any increase in heron abundance, since no nesting occurs during this period. However, post-reproductive molting by great blue herons peaks in September and October [26], and may account for the apparent increase in late-season feeding on this host by mosquitoes [11]. Most telling is the increase in frog-feeding during the calling season. In frogs, increased population density associated with reproduction is not realized until months later, when aquatic tadpoles transform and become available to mosquitoes. The increased incidence of frog blood meals that we document during times of increased calling of male frogs occurs when frog abundance is generally unchanged except for the redistribution of adults during reproduction. Although the increase in abundance of each host during periods of hatching/birthing likely contributes to increased host use during the reproductive phase, our data and those of other studies [3], [27] suggest that host abundance alone cannot explain seasonal patterns of host use.

This work constitutes the first study to examine changes in species-specific host preference over time. Many other studies [28], [29], [30] have examined host preference of mosquitoes, however these studies either failed to incorporate the effects of seasonal changes in host use (host use data pooled from all seasons), or examined patterns of host preference at taxonomic levels above that of species (usually order or class [3]). A recent meta-analysis found that feeding patterns of the mosquito community are more a function of host availability than innate mosquito preference for any given host [31]. Host abundance and host defensive behavior are considered to be the two main components of host availability [31]. Our results suggest that defensive behaviors, (or their absence), as dictated by reproductive phenology, may be even more important than host abundance in determining host availability and therefore, patterns of host use by mosquitoes. Here, we demonstrate that patterns of blood-feeding can change dramatically over relatively brief time periods and that seasonal patterns of host use by mosquitoes reflect the reproductive phenology of the host animals that are associated with changes in host defensive behaviors. These results contribute to our understanding of the biological factors which underlie seasonal patterns of host use which, in turn, drive epidemics of human disease.

## Materials and Methods

### Determination of mosquito host use

Host use data was obtained by blood-meal identification of mosquitoes aspirated from resting sites [32] within Tuskegee National Forest, AL and surrounding privately-owned lands. The field site encompassed a 28 km^2^ circle, with a variety of habitat types, including beaver ponds, rush marsh, oxbow lakes, hardwood swamp, upland pine forest, mixed hardwood and coniferous forest, and hardwood bottomland [33]. Sampling locations (resting sites) were scattered throughout the study area and throughout each habitat type to obtain representative samples from the spectrum of available mosquito and host species. Mosquitoes were aspirated weekly from natural (vegetation, animal burrows, and hollow trees) and man-made resting sites (resting boxes, fiber pots, and garbage cans) [32] during months with adult mosquito activity (February - October) over seven years (2001–2004, 2006–2008). Individual blood-engorged female mosquitoes were subjected to PCR-based assays targeting the vertebrate cytochrome B gene to identify the source of vertebrate blood [34], [35].

### Reproductive phenology of host animals

For the most commonly fed upon hosts, we collected data on various aspects of reproductive biology, including nesting phenology, birth dates, and male vocalizations for mate attraction. White-tailed deer (*Odocoileus virginianus*) fawning dates were obtained from herds on managed lands within 65 km of the mosquito collection site (2002–2003, 2005–2008). Alabama Division of Wildlife and Freshwater Fisheries personnel collected female deer using firearms and determined age (days) of fetuses, used to estimate date of parturition [36]. Cumulative births (number of available fawns) per semimonthly period were used in the analysis. The timing of great blue heron (*Ardea herodias*) nesting was determined by semiweekly observations of a heron rookery within the study site (2008–2009). Adult, juvenile and nestling herons observed in the rookery were counted at each visit until the rookery was abandoned each year (late June). Counts were then used to calculate the number of individuals occupying the rookery per semimonthly period. Nesting data for yellow-crowned night-heron, (*Nyctanassa violacea*), were obtained from the Cornell Lab of Ornithology, North American Nest-Record Card Program. The predicted number of chicks per nest per semimonthly period was calculated from historic nesting records from six southeastern states (Florida (northern counties only), Georgia, Louisiana, South Carolina, Tennessee and Texas) from 1976 to 1995. No records from Alabama were available. The presence of calling male frogs (males attracting mates) was determined from monthly visits (February – October) to five ponds within the study site in 2008 [37]. Detection/non-detection data were then used to model monthly detection probabilities using general linear mixed models [38] with a binomial distribution and a logit-link. Models were built using a stepwise procedure in which non-significant predictor variables (p > 0.05) were sequentially removed [39]. Candidate variables included linear and squared terms for the month in which the observation was made. Modeling was performed using R [40]. Resulting monthly detection probabilities were used to categorize each frog species as either early breeder or late breeder. Mean detectability was then calculated for each breeding cohort. Frog species that were not detected in mosquito blood meals and which showed no significant changes in detectability across months were not used in the analysis. Months with fewer than five frog-derived blood meals (September and October) were not used in the analysis.

### Statistical analysis

We used linear mixed-effects models fit using maximum likelihood to investigate the relationship between relative host use and host reproductive biology. Relative host use was calculated as the proportion of total blood meals originating from a given host in a given time period (semimonthly or monthly, depending on the host group). Blood meal data for each host species were summed across years prior to calculation of relative host use. All proportions were arcsine square root transformed (angular transformation) prior to analysis. For each host we fit a model containing a fixed effect for the host reproduction variable and a null model which assumed constant host use across time. In all models, we included a random intercept for the time step of each observation to control for possible autocorrelation among the data points. Models were ranked and compared using Akaike's Information Criterion corrected for small sample sizes (AIC_c_) [41]. Analyses were performed using R [40].

For white-tailed deer, great blue heron, and yellow-crowned night heron, data were analyzed in semimonthly increments. Data from 1,378 blood meal identifications from six mosquito species (Table S1) were included in the analysis (*Culex erraticus, Culex peccator, Aedes vexans, Culex quinquefasciatus, Coquillettidia perturbans* and *Aedes sticticus*). Species of *Anopheles*, which took >90% of blood meals from a single host species (white-tailed deer), were not included in the analyses.

For anuran hosts, relative host use was calculated as the proportion of total blood meals each month derived from early- or late-breeding frogs. Data from 164 frog-derived blood meal identifications (Table S1) from two mosquito species (*Culex territans* and *Culex peccator*) were included in the analysis for anuran hosts. No other mosquito species commonly fed on frogs.

## Supporting Information

Table S1 **Hosts of** ***Aedes sticticus, Aedes vexans, Coquillettidia perturbans, Culex erraticus, Culex peccator, Culex quinquefasciatus*** **and** ***Culex territans*** **from Tuskegee National Forest, AL, USA (2001–2004 and 2006–2008).** Host use was determined by PCR-based assays identifying the vertebrate source of blood from field-collected mosquitoes.
(DOC)
