# Supplementary material for: Host Reproductive Phenology Drives Seasonal Patterns of Host Use in Mosquitoes
Source: PLoS One. 2011 Mar 7;6(3):e17681. doi: 10.1371/journal.pone.0017681 (PMC3049777; doi:10.1371/journal.pone.0017681)
Supplement: Table S1 — Hosts of Aedes sticticus, Aedes vexans, Coquillettidia perturbans, Culex erraticus, Culex peccator, Culex quinquefasciatus and Culex territans from Tuskegee National Forest, AL, USA (2001–2004 and 2006–2008). Host use was determined by PCR-based assays identifying the vertebrate source of blood from field-collected mosquitoes. (DOC) [file pone.0017681.s001.doc]

Table S1. Hosts of *Aedes sticticus, Aedes vexans, Coquillettidia perturbans, Culex erraticus, Culex peccator, Culex quinquefasciatus* and *Culex territans* from Tuskegee National Forest, AL, USA (2001-2004 and 2006-2008). Host use was determined by PCR-based assays identifying the vertebrate source of blood from field-collected mosquitoes.

|  |  |  | Mosquito species | | |  |  |  |
| --- | --- | --- | --- | --- | --- | --- | --- | --- |
| Host common name | *Aedes*  *sticticus* | *Aedes*  *vexans* | *Coquillettidia*  *perturbans* | *Culex*  *erraticus* | *Culex*  *peccator* | *Culex*  *quinquefasciatus* | *Culex*  *territans* | Total |
| Acadian flycatcher |  |  |  | 1 |  |  |  | 1 |
| American beaver |  |  |  | 3 |  |  |  | 3 |
| American bittern |  |  |  | 21 | 1 |  |  | 22 |
| American robin |  |  |  | 14 |  | 1 |  | 15 |
| Anhinga |  |  |  | 2 |  |  |  | 2 |
| Barking treefrog |  |  |  |  |  |  | 3 | 3 |
| Barred owl |  |  |  | 18 |  |  |  | 18 |
| Bird-voiced treefrog |  |  |  |  |  |  | 18 | 18 |
| Black rat snake |  |  |  | 5 |  |  |  | 5 |
| Blue grosbeak |  |  |  | 2 |  |  |  | 2 |
| Blue jay |  |  |  | 3 |  |  |  | 3 |
| Bullfrog |  |  |  | 1 | 26 |  | 13 | 40 |
| Canebrake rattlesnake | |  |  | 1 | 1 |  |  | 2 |
| Carolina chickadee |  |  | 1 | 17 |  |  |  | 18 |
| Carolina wren |  |  |  | 1 |  |  |  | 1 |
| Common grackle |  | 2 |  | 4 |  |  |  | 6 |
| Cope's grey tree frog | |  |  | 1 |  |  | 18 | 19 |
| Cottonmouth |  |  |  | 7 | 76 |  |  | 83 |
| Cow |  |  |  | 5 |  |  |  | 5 |
| Dog |  |  |  | 3 |  |  |  | 3 |
| Eastern box turtle |  |  |  | 3 |  |  |  | 3 |
| Eastern cottontail |  |  |  | 5 | 1 |  |  | 6 |
| Eastern fence lizard |  |  |  | 2 |  |  |  | 2 |
| Eastern racer |  |  |  | 6 | 1 |  |  | 7 |
| Eastern ribbon snake |  |  |  | 7 | 3 |  |  | 10 |
| Eastern screech owl |  |  |  | 3 |  |  |  | 3 |
| Eastern wood rat |  |  |  | 1 |  |  |  | 1 |
| Garter snake |  |  |  | 1 |  |  |  | 1 |
| Glossy crayfish snake |  |  |  | 1 |  |  |  | 1 |
| Gray jungle fowl |  |  |  | 1 |  |  |  | 1 |
| Gray squirrel |  |  |  |  |  | 1 |  | 1 |
| Great blue heron |  | 1 |  | 140 | 9 |  |  | 150 |
| Great egret |  |  |  | 1 |  |  |  | 1 |
| Green heron |  |  |  | 17 | 1 |  |  | 18 |
| Green anole |  |  |  |  | 2 |  |  | 2 |
| Green frog |  |  |  |  | 13 |  | 4 | 17 |
| Green heron |  |  |  | 8 |  |  |  | 8 |
| Green tree frog |  |  |  |  | 1 |  | 27 | 28 |
| Grey catbird |  |  |  | 3 |  |  |  | 3 |
| Hooded warbler |  |  |  | 1 |  |  |  | 1 |
| House finch |  |  | 1 | 2 |  | 1 |  | 4 |
| Human |  |  |  | 31 | 5 |  |  | 36 |
| Kentucky warbler |  |  |  | 1 |  |  |  | 1 |
| Leopard frog |  |  |  |  | 3 |  | 9 | 12 |
| Northern cardinal | 1 | 3 |  | 22 |  | 4 |  | 30 |
| Northern mockingbird |  |  |  | 12 |  | 1 |  | 13 |
| Orchard oriole |  |  |  | 2 |  |  |  | 2 |
| Pied-billed grebe |  | 1 |  | 7 |  |  |  | 8 |
| Pine warbler |  |  |  | 1 |  |  |  | 1 |
| Pine woods treefrog |  |  |  | 1 |  |  | 2 | 3 |
| Plain-bellied water snake | |  |  | 9 | 7 |  |  | 16 |
| Raccoon | 1 |  |  | 9 |  |  |  | 10 |
| Red-eared slider |  |  |  | 1 |  |  |  | 1 |
| Red-tailed hawk |  |  |  |  |  | 1 |  | 1 |
| Ruby-throated hummingbird | |  |  | 1 |  |  |  | 1 |
| Southern toad |  |  |  |  |  |  | 1 | 1 |
| Spring peeper |  |  |  |  |  |  | 21 | 21 |
| Squirrel treefrog |  |  |  |  |  |  | 2 | 2 |
| Summer tanager |  |  |  | 1 |  |  |  | 1 |
| Swamp rabbit | 1 | 1 |  | 6 |  |  |  | 8 |
| Tufted titmouse |  |  |  | 6 |  |  |  | 6 |
| White-eyed vireo |  | 1 |  | 5 |  |  |  | 6 |
| White-tailed deer | 1 | 22 | 7 | 632 | 2 | 1 |  | 665 |
| Wild boar |  |  |  | 1 | 1 |  |  | 2 |
| Wild turkey |  | 2 |  | 4 |  |  |  | 6 |
| Wood duck |  |  |  | 16 | 1 |  |  | 17 |
| Wood thrush |  |  |  | 2 |  | 1 |  | 3 |
| Yellow-billed cuckoo |  |  |  | 4 |  |  |  | 4 |
| Yellow-crowned night heron | |  |  | 71 | 1 |  |  | 72 |
| Grand total | 4 | 33 | 9 | 1162 | 158 | 12 | 118 | 1486 |
